# Risk and diagnostic factors and therapy outcome of neonatal early onset sepsis in ICU patients of Saudi Arabia: a systematic review and meta analysis

**DOI:** 10.3389/fped.2023.1206389

**Published:** 2023-08-23

**Authors:** Mohammed K. Alshammari, Ahlam H. Alsanad, Rawan J. Alnusayri, Abdulmajeed S. Alanazi, Fatmah Q. Shamakhi, Khaled M. Alshahrani, Abdullah M. Alshahrani, Ghaliah Yahya, Abdulaziz A. Alshahrani, Turki S. Alshahrani, Hamad S. Sultan, Fatimah M. Alshahrani, Fouzyia A. Alreshidi, Renad A. Alnigaidan, Abdulaziz A. Almazyad

**Affiliations:** ^1^ Department of Clinical Pharmacy, King Fahad Medical City Hospital, Riyadh, Saudi Arabia; ^2^ Department of Neonatal Intensive Care Unit, Maternity and Children Hospital, Dammam, Saudi Arabia; ^3^ Department of Pharmacy, Maternity and Children Hospital, Ministry of Health, Jouf, Saudi Arabia; ^4^ Department of Medicine, Ministry of Health, Abha, Saudi Arabia; ^5^ Department of Medicine, Ministry of Health, Khamis Mushait, Saudi Arabia; ^6^ Department of Medicine, King Khaled University, Abha, Saudi Arabia; ^7^ College of Medicine, King Khalid University, Al Fara, Saudi Arabia; ^8^ Department of Pharmacy, Dr. Sulaiman Al-Habib Hospital, Khobar, Saudi Arabia; ^9^ Department of Nursing Office, Hail General Hospital, Hail, Saudi Arabia; ^10^ Department of Pharmacy, Al-Dawaa Medical Services Company Limited, Unaizah, Saudi Arabia; ^11^ Department of Pharmacy, Qassim University Medical City Hospital, Buraidah, Saudi Arabia

**Keywords:** risk factors, diagnostic factors, therapy outcome, neonatal early onset sepsis, ICU patients, Saudi Arabia

## Abstract

**Background:**

Neonatal early onset sepsis (NEOS) is a serious and potentially life-threatening condition affecting newborns within the first few days of life. While the diagnosis of NEOS was based on clinical signs and symptoms in the past, recent years have seen growing interest in identifying specific diagnostic factors and optimizing therapy outcomes. This study aims to investigate the diagnostic and risk factors and therapy outcomes of neonatal EOS in ICU patients in Saudi Arabia, with the goal of improving the management of neonatal EOS in the country.

**Methods:**

This method outlines the protocol development, search strategy, study selection, and data collection process for a systematic review on neonatal early onset sepsis in Saudi Arabian ICU patients, following the PRISMA 2020 guidelines. PRISMA (Preferred Reporting Items for Systematic Reviews and Meta-Analyses) is a well-established guideline that provides a framework for conducting systematic reviews and meta-analyses in a transparent and standardized manner. It aims to improve the quality and reporting of such research by ensuring clear and comprehensive reporting of study methods, results, and interpretations. The search strategy included electronic databases (PubMed, Embase, Google Scholar, Science Direct, and the Cochrane Library) and manual search of relevant studies, and data were extracted using a standardized form.

**Results:**

The systematic review included 21 studies on neonatal sepsis in Saudi Arabia, with varying study designs, sample sizes, and prevalence rates of sepsis. Group B streptococcus and E. coli were the most commonly isolated pathogens. Various diagnostic factors and risk factors were reported, including hematological parameters, biomarkers, and blood cultures. The quality of the included studies was assessed using the Newcastle-Ottawa Scale and Joanna Briggs Institute critical checklist.

**Conclusions:**

The review identified a number of risk and diagnostic factors and therapy outcomes for neonatal sepsis. However, most of the studies were having small scale cohort groups. Further research with controlled study designs is needed to develop effective prevention and management strategies for neonatal sepsis in Saudi Arabia.

## 1. Introduction

Neonatal early onset sepsis (NEOS) is a serious and potentially life-threatening condition that affects newborns within the first few days of life (1, 2). It is characterized by systemic infection and inflammation, which can lead to septic shock, multiple organ failure, and death if not promptly and effectively treated (3, 4). The pathophysiology predominantly involves ascending colonisation of the uterine cavity and maternal vaginal tract by the typical bacterial flora of the genitourinary and gastrointestinal tracts, which leads to eventual colonisation and infection of the foetus or newborn (5). NEOS is a major health concern worldwide, with an estimated incidence of 0.5–1.5 cases per 1,000 live births in developed countries, and up to 5 cases per 1,000 live births in low- and middle-income countries (LMICs) (6, 7). In 3%–4% of newborns who have NEOS, there may be serious systemic disease and even deaths are possible outcomes (8, 9).

In the past, the diagnosis of NEOS was based on clinical signs and symptoms, such as fever, lethargy, poor feeding, and respiratory distress, and was often treated empirically with broad-spectrum antibiotics (1, 10, 11). However, this approach led to overuse of antibiotics, increased healthcare costs, and the emergence of antibiotic-resistant bacteria (12). In recent years, there has been growing interest in identifying specific diagnostic factors that can accurately predict the presence or absence of NEOS, and in optimizing therapy outcomes through targeted antibiotic therapy and supportive measures (13, 14). The risk factors for NEOS include maternal factors such as maternal fever, chorioamnionitis, and prolonged rupture of membranes, as well as neonatal factors such as low birth weight, prematurity, and invasive procedures (2, 4, 15–17). In addition, laboratory parameters such as elevated C-reactive protein (CRP) levels, low platelet count, and abnormal white blood cell (WBC) count have been shown to be useful in predicting the presence of NEOS (13, 14).

Despite the availability of diagnostic tools and risk stratification algorithms, the management of neonatal EOS remains challenging, particularly in neonatal intensive care unit (NICU) patients (18). Guidelines for the prevention of NEOS are provided by the Centers for Disease Control and Prevention (CDC), the American Academy of Pediatrics, and the American Congress of Obstetricians and Gynecologists, and provide algorithms for the diagnosis and management of high risk NEOS patients (19, 20). These recommendations are based on epidemiological data collected prior to the widespread use of intrapartum antibiotic prophylaxis in obstetrics, when NEOS incidence was 5–10-fold greater than presently found (21–23). These recommendations lead to a significant number (15%–20%) of term and late preterm babies being examined for sepsis and getting 5%–8% of empirical antibiotics (24). The optimal duration of antibiotic therapy, the choice of antibiotics, and the use of adjunctive therapies such as intravenous immunoglobulin (IVIG) and granulocyte-colony stimulating factor (G-CSF) remain topics of debate (25, 26).

Saudi Arabia is a high-income country with a well-established healthcare system, including specialized NICUs. However, the incidence and outcomes of neonatal EOS in Saudi Arabia, along with other Gulf countries, remain insufficiently characterized (27, 28). Nevertheless, NEOS is a major health concern in neonatal intensive care units (NICUs) in Saudi Arabia. Identifying diagnostic factors and optimizing therapy outcomes are crucial for the management of EOS in these patients. This study aims to investigate the diagnostic factors and therapy outcomes of neonatal EOS in ICU patients in Saudi Arabia. The study explores the clinical presentation of EOS, laboratory parameters, and risk factors associated with the development of EOS. The findings of this study will contribute to improving the management of neonatal EOS in Saudi Arabia and provide insight into optimizing therapy outcomes for this vulnerable patient population.

## 2. Materials and methods

### 2.1. Protocol development

The methodology for this study on followed the PRISMA (Preferred Reporting Items for Systematic Reviews and Meta-Analyses) 2020 guidelines to ensure transparent reporting and reduce bias in the study. PRISMA is a well-established guideline that provides a framework for conducting systematic reviews and meta-analyses in a transparent and standardized manner. It aims to improve the quality and reporting of such research by ensuring clear and comprehensive reporting of study methods, results, and interpretations. The study protocol was developed before the commencement of the study, outlining the research question, search strategy, inclusion and exclusion criteria, data collection and analysis methods, and ethical considerations.

### 2.2. Search strategy

A comprehensive search of electronic databases such as PubMed, Embase, Google Scholar, Science Direct, and the Cochrane Library was conducted to identify studies relevant to the research question. The search included keywords such as “neonatal sepsis,” “early onset sepsis,” “ICU patients,” “diagnostic factors,” “risk factors,” and “therapy outcomes.” The search strategy also include MeSH terms and keywords related to neonatal EOS, ICU patients, and Saudi Arabia. A manual search of the reference lists of relevant studies was also performed. The search strategy was designed by two authors who are expert in this field and reviewed by another research team among co-authors. The details are given in Supplementary Table S1 as supplementary file.

### 2.3. Study selection

Two independent reviewers screened the titles and abstracts of the identified studies to determine their eligibility for inclusion. The full texts of potentially relevant studies were assessed for eligibility based on predefined inclusion and exclusion criteria. Studies that meet the following criteria were included in the study: (1) conducted in Saudi Arabia, (2) neonates diagnosed with early onset sepsis, (3) admitted to ICU, (4) diagnostic and risk factors and therapy outcomes reported, and (5) observational or interventional studies. Studies that did not meet the inclusion criteria were excluded. Any discrepancies between the reviewers were resolved through discussion and consensus.

### 2.4. Data collection

Data were extracted from the eligible studies using a standardized data extraction form. The data in Table 1 included information on author and year, location, study period and settings, NEOS definition, study design, sample size and microbiological data on common causative pathogens. In Table 2, information was included related to diagnostic factors (hematological parameters, biomarkers, blood cultures), mother-related risk factors (premature ruptures of membranes, gestational age at delivery, mode of delivery and delivery <37 weeks of gestation), neonate-related risk factor (low apgar score, resuscitation at birth, need for artificial ventilation, low birth weight) and therapy outcome parameters (length of nicu stay, recovery/success rate, change of antibiotics and death). This review was performed between January and April 2023.

**Table 1:** Characteristics of included studies.

Author and year	Location	Study period	Settings	Neonatal definition	Study design	Sample Size	Top 3 isolated pathogens		
Al-Matary et al., 2019 (27)	Riyadh	January 2011–December 2015	King Fahad Medical City Hospital	<72 h	Retrospective study	245	Group B streptococcus (33.3%)	*E. coli* (27.3%)	*Streptococcus* species (12.1%)
Al-Mouqdad et al., 2018 (29)	Riyadh	July 2015–June 2017	King Saud Medical City	Not stated	Retrospective study	295	–	–	–
Al-Rafiaah et al., 2016 (30)	Riyadh	2014	King Abdul Aziz Medical City	Not stated	Retrospective study	85	*E. coli* (29.4%)	Coagulase negative Staphylococcus (23.5%)	Group B streptococcus (17.6%)
Al-Zaharani et al., 2015 (31)	Taif	January 2013–January 2014	King Abdul Aziz hospital	Not stated	Retrospective study	100	Klebsiella spp. (28.0%)	*E. coli* (20.0%)	Group B streptococcus (12.0%)
Almuneef et al., 2000 (32)	Riyadh	January 1990–December 1994	King Fahad National Guard hospital	<7 days	Retrospective study	23	Group B streptococcus (100%)	–	–
Hammoud et al., 2017 (28)	Taif	June 2013- May 2015	King Abdul Aziz hospital Maternity and children's hospital	<72 h	Prospective study	30,389	Group B streptococcus (59.8%)	*E. coli* (12.7%)	Coagulase negative Staphylococcus (5.9%)
Kilani et al., 2000 (33)	Riyadh	January 1996–December 1997	King Khalid University Hospital	<48 h	Retrospective study	865	*E. coli* (28.6%)	*MSSA* (21.4%)	Coagulase negative Staphylococcus (14.3%)
Huseynova et al., 2021 (34)	Riyadh	February 2020–June 2020	King Saud Medical City	<72 h	Retrospective study	44	Group B streptococcus (6.8%)	–	–
Al-Mudeer et al., 2020 (35)	Jazan	May 2012–April 2019	King Fahad Central Hospital	<72 h	Retrospective study	28,337	*E. coli* (29%)	Group B streptococcus (17%)	Coagulase negative Staphylococcus (11%)
Luhidan et al., 2019 (36)	Riyadh	2004–2014	King Abdullah Specialized hospital	<7 days	Retrospective study	55	Group B streptococcus (100%)	–	–
Sobaih et al., 2014 (37)	Riyadh	January 1999–December 2007	King Khalid University Hospital	<72 h	Retrospective study	468	*Staphylococcus epidermidis* (45%)	*Staphylococcus aureus* (11.8%)	*Klebsiella pneumoniae* (11.8%)
Nasr et al., 2013 (38)	Taif	March 2012–August 2012	King Abdul Aziz hospital	Not stated	Prospective study	205	–	–	–
Fattah et al., 2017 (39)	Riyadh	January 2013–August 2015	King Abdul Aziz Medical City	Not stated	Prospective study	320	*E. coli* (68.0%)	*H. influenzae* (27.0%)	Group B streptococcus (6.0%)
Dawodu et al., 1997 (40)	Khobar	September 1983–September 1988	King Fahd Hospital	<48 h	Case-control	61	*Staphylococcus epidermidis* (18.0%)	*Klebsiella Enterobacter Serratia* (8.1%)	*S aureus* (3.2%)
Barnawi et al., 2020 (41)	Madina	March 2019–May 2019	Madina Maternity and Children hospital	Not stated	Case study	1	*Elizabethkingia meningoseptica* (100%)	–	–
Haque et al., 1990 (42)	Riyadh	October 1983–July 1988	King Khalid University Hospital	<48 h	Retrospective study	190	*Staphylococcus epidermidis* (35.7%)	*S aureus* (17.3%)	*Klebsiella* spp. (9.4%)
Ohlsson et al., 1986 (43)	Riyadh	November 1980–October 1984	King Faisal Specialist hospital	<48 h	Retrospective study	49	*Klebsiella Enterobacter Serratia* (21.3%)	*E. coli* (17.6%)	*S. aureus* (15.4%)
Alsohime et al., 2019 (44)	Riyadh	–	King Saud Medical City	Not stated	Case study	1	Actinomyces neuii (100%)	–	–
Elbashier et al., 1994 (45)	Qatif	January 1989–January 1992	Qatif Central hospital	<48 h	Prospective study	144	*Klebsiella* spp. (29.7%)	*E. coli* (21.6%)	*Pseudomonas* spp. (5.4%)
Abu-Osba et al., 1989 (46)	Dhahran	January 1981–December 1986	Dhahran Health Center	<3 days	Retrospective study	76	*Staphylococcus epidermidis* (44.4%)	Enterococcus (33.3%)	*E. coli* (30.0%)
Srair et al., 1993 (47)	Qatif	June 1992–May 1993	Qatif Central hospital	<72 h	Prospective study	53	*E. coli* (15.1%)	Group B streptococcus (11.3%)	*Streptococcal faecalis* (9.4%)

**Table 2:** Diagnostic and risk factors and therapy outcomes of NEOS.

Author and year	Diagnostic factors			Risk factors								Therapy outcomes			
				Mother-related				Neonate-related							
	Hematological parameters	Biomarkers	Blood cultures	Premature ruptures of membranes	Gestational age at delivery	Mode of delivery	Delivery <37 weeks of gestation	Low Apgar score	Resuscitation at birth	Need for artificial ventilation	Low birth weight	Length of NICU stay	Recovery/success rate	Change of antibiotics	Death
Al-Matary et al., 2019 (27)															
Al-Mouqdad et al., 2018 (29)															
Al-Rafiaah et al., 2016 (30)															
Al-Zaharani et al., 2015 (31)															
Almuneef et al., 2000 (32)															
Hammoud et al., 2017 (28)															
Kilani et al., 2000 (33)															
Huseynova et al., 2021 (34)															
Al-Mudeer et al., 2020 (35)															
Luhidan et al., 2019 (36)															
Sobaih et al., 2014 (37)															
Nasr et al., 2013 (38)															
Fattah et al., 2017 (39)															
Dawodu et al., 1997 (40)															
Barnawi et al., 2020 (41)															
Haque et al., 1990 (42)															
Ohlsson et al., 1986 (43)															
Alsohime et al., 2019 (44)															
Elbashier et al., 1994 (45)															
Abu-Osba et al., 1989 (46)															
Srair et al., 1993 (47)															
Total	10	7	18	5	5	7	7	5	1	2	12	3	8	5	11

### 2.5. Meta-analysis

The statistical analysis was performed using RStudio software (RStudio Team, Boston, MA; version R, 2023.03.0) using Meta Package and Metaprop command. The proportion test was performed to estimate the proportion of risk factors. I-squared (*I*^2^) index statistic was employed to evaluate the assertiveness heterogeneity in choice of effects, considering the randomized effect for the analysis. A threshold of 0.05 was considered significant.

## 3. Results

### 3.1. Included studies

**Figure 1:**
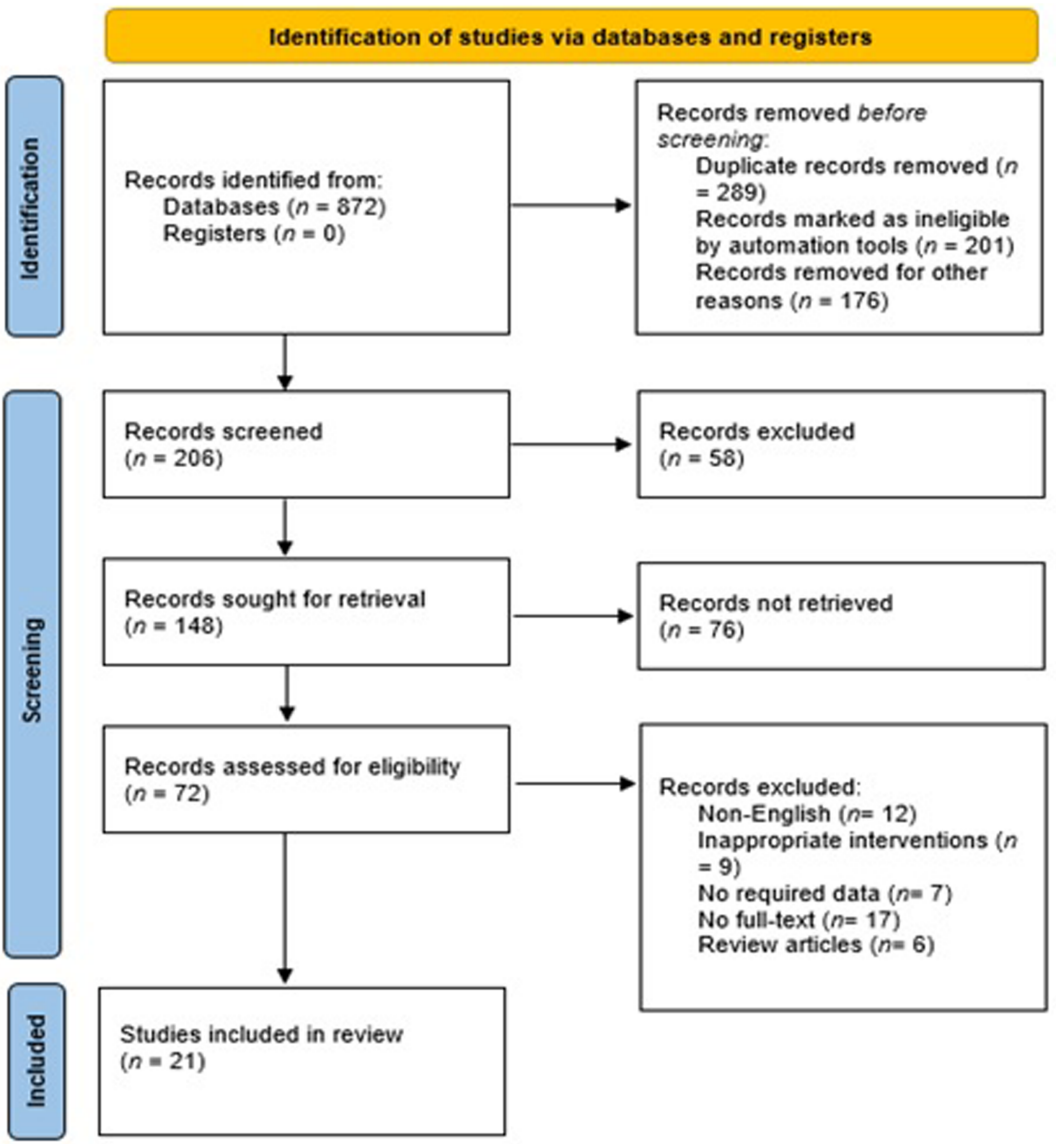
Flowchart of included studies.

**Figure 2:**
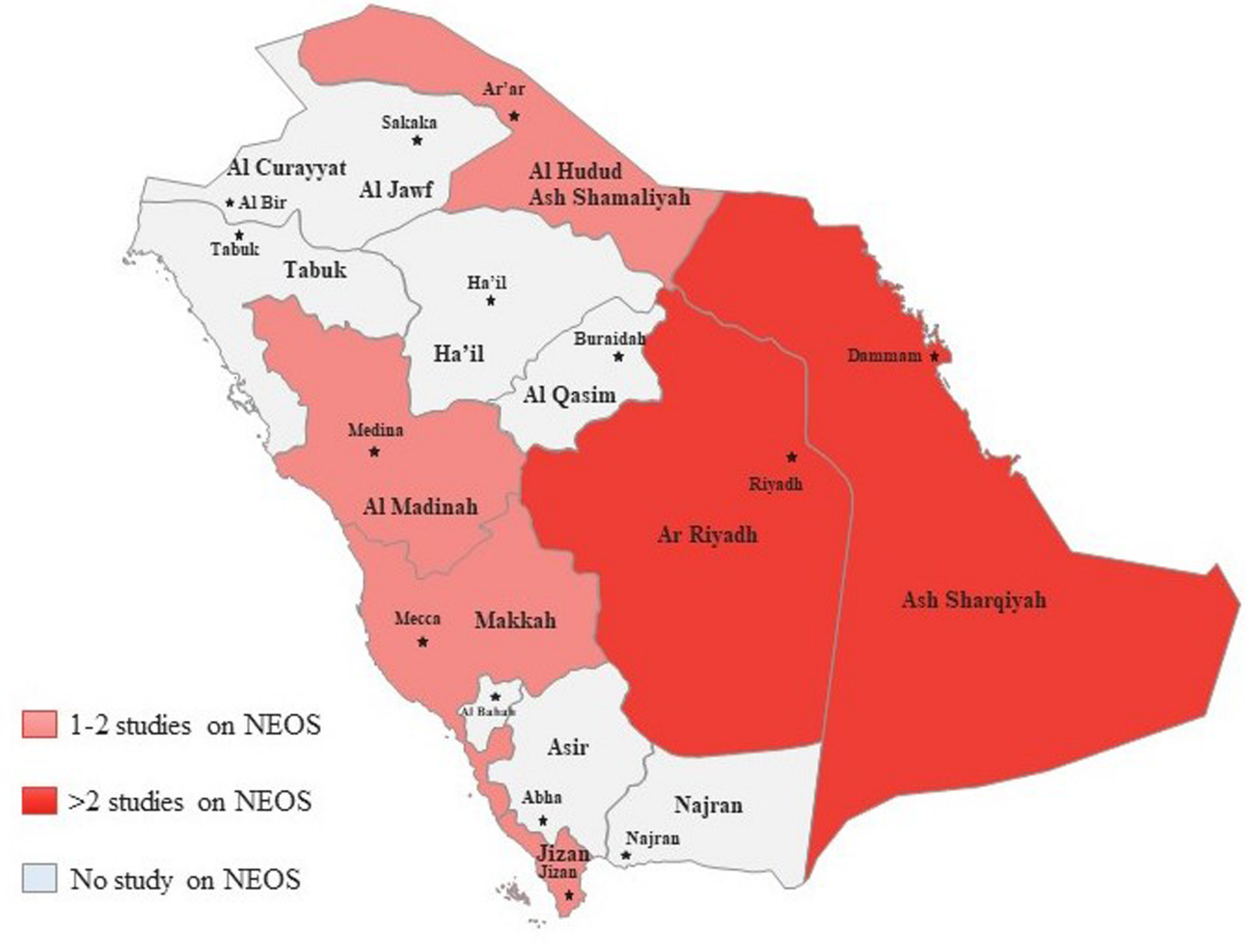
Numbers of studies in different provinces of KSA.

The PRISMA 2020 flow diagram is shown in Figure 1 which is a graphical representation of the systematic review process. It outlines the steps involved in identifying, screening, and selecting studies for inclusion in a review. In this particular study, the review included searches of databases only. The diagram starts with the identification of studies via databases and registers, where 872 records were identified from databases and none from registers. After identifying the records, the next step was to remove duplicate records, which accounted for 289 records. In addition, 201 records were marked as ineligible by automation tools, and 176 records were excluded based on the initial screening of titles and abstracts. These studies did not meet the predefined inclusion criteria for our systematic review. The reasons for exclusion at this stage included irrelevant topics, unrelated interventions, and studies conducted in settings other than Saudi Arabia. Of the remaining 206 screened records, 58 were excluded further during the full-text assessment phase. These exclusions were due to the identification of commentaries, guidelines, and book chapters, which were not eligible for inclusion in our systematic review. The remaining 148 records were sought for retrieval, but 76 records were not retrieved. The 72 retrieved records were assessed for eligibility, where 12 non-English studies, 9 studies with inappropriate interventions, 7 studies with no required data, 17 studies with no full-text, and 6 review articles were excluded. Finally, 21 studies were included in the review. These studies met the eligibility criteria and were included in the systematic review. Figure 2 showed that most of the studies were conducted from Riyadh and eastern provinces of KSA.

**Figure 3:**
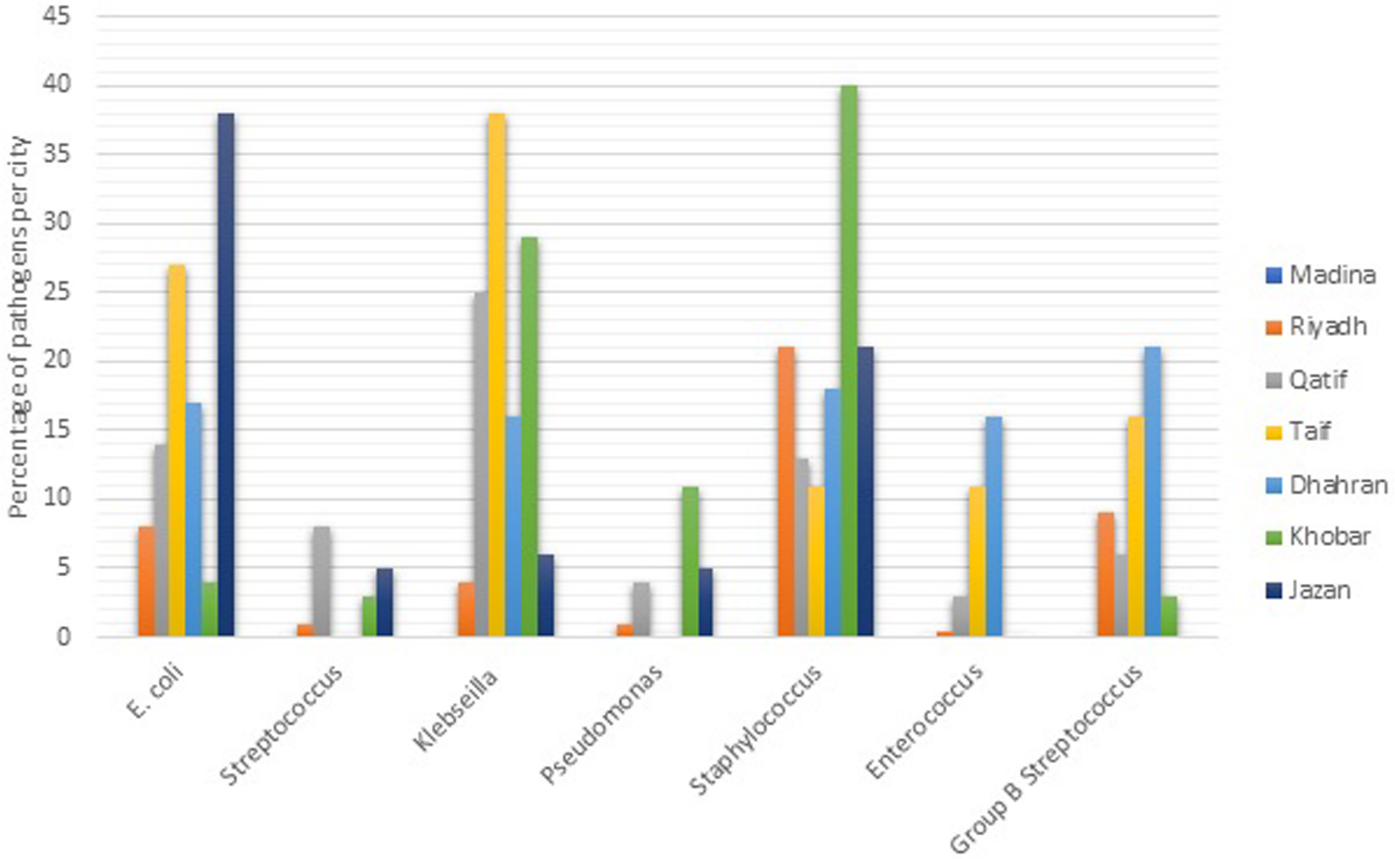
Distribution of pathogens in different cities of KSA.

The studies used different study designs, including retrospective, prospective, case-control, and case studies, and varied in their neonatal definitions of NEOS, study period, and sample sizes. The Table 1 included the results of 21 different studies on neonatal sepsis conducted in different locations and settings in Saudi Arabia. The studies varied in terms of study design, sample size, and prevalence of neonatal sepsis (NOS). The sample sizes of the studies varied widely, ranging from 1 in case study to 30,389 in prospective study. The larger sample sizes were reported by Al-Mudeer et al. (2020) and Hammoud et al. (2017), both of which had over 28,000 participants. The studies were conducted in different settings, including medical centers, hospitals, and maternity and children's hospitals. The top three isolated pathogens varied across the studies. Group B streptococcus (GBS) and E. coli were the most commonly isolated pathogens, with GBS being the most commonly isolated pathogen in 5 of the 21 studies. Other pathogens that were commonly isolated include Coagulase negative Staphylococcus (CNS), Klebsiella spp., and *Staphylococcus epidermidis*. The distribution of pathogens in different cities of KSA is presented in Figure 3.

The present study also aimed to investigate the diagnostic and risk factors and therapy outcomes of NEOS in ICU patients in Saudi Arabia as shown in Table 2. The analysis of the results obtained from the 21 selected studies revealed that various factors were used for the diagnosis of EOS, including hematological parameters, biomarkers and blood cultures, The use of hematological parameters, such as white blood cell count, absolute neutrophil count, and immature to total neutrophil ratio, was reported by some studies as a diagnostic factor for EOS. In contrast, biomarkers such as C-reactive protein (CRP), procalcitonin (PCT), and interleukin-6 (IL-6) were reported by several studies as useful tools for the diagnosis of EOS. Blood cultures are considered the gold standard for the diagnosis of NEOS and several studies reported that blood cultures were used as a diagnostic factor for NEOS. Regarding the mother and neonate related risk factors, gestational age at delivery was reported as a significant risk factor for NEOS by some studies. Preterm delivery is known to increase the risk of NEOS due to the immature immune system of preterm infants. Moreover, premature rupture of membranes was reported by some studies as a significant risk factor for EOS. Low Apgar score at birth, need for artificial ventilation, and low birth weight were also reported by some studies as significant risk factors for NEOS. The length of NICU stay was reported as a neonate-related risk factor by some studies. Mode of delivery including vaginal delivery and caesarean section was reported by some studies as a significant risk factor for NEOS. In terms of therapy outcomes, the recovery or success rate of therapy was reported by several studies. The change of antibiotics during treatment was reported by some studies. The overall death rate was reported by most of the studies. Nevertheless, the present study revealed that there is heterogeneity among the diagnostic factors and risk factors reported by the selected studies. This heterogeneity may be due to differences in the study population.

### 3.2. Meta-analysis

**Figure 4:**
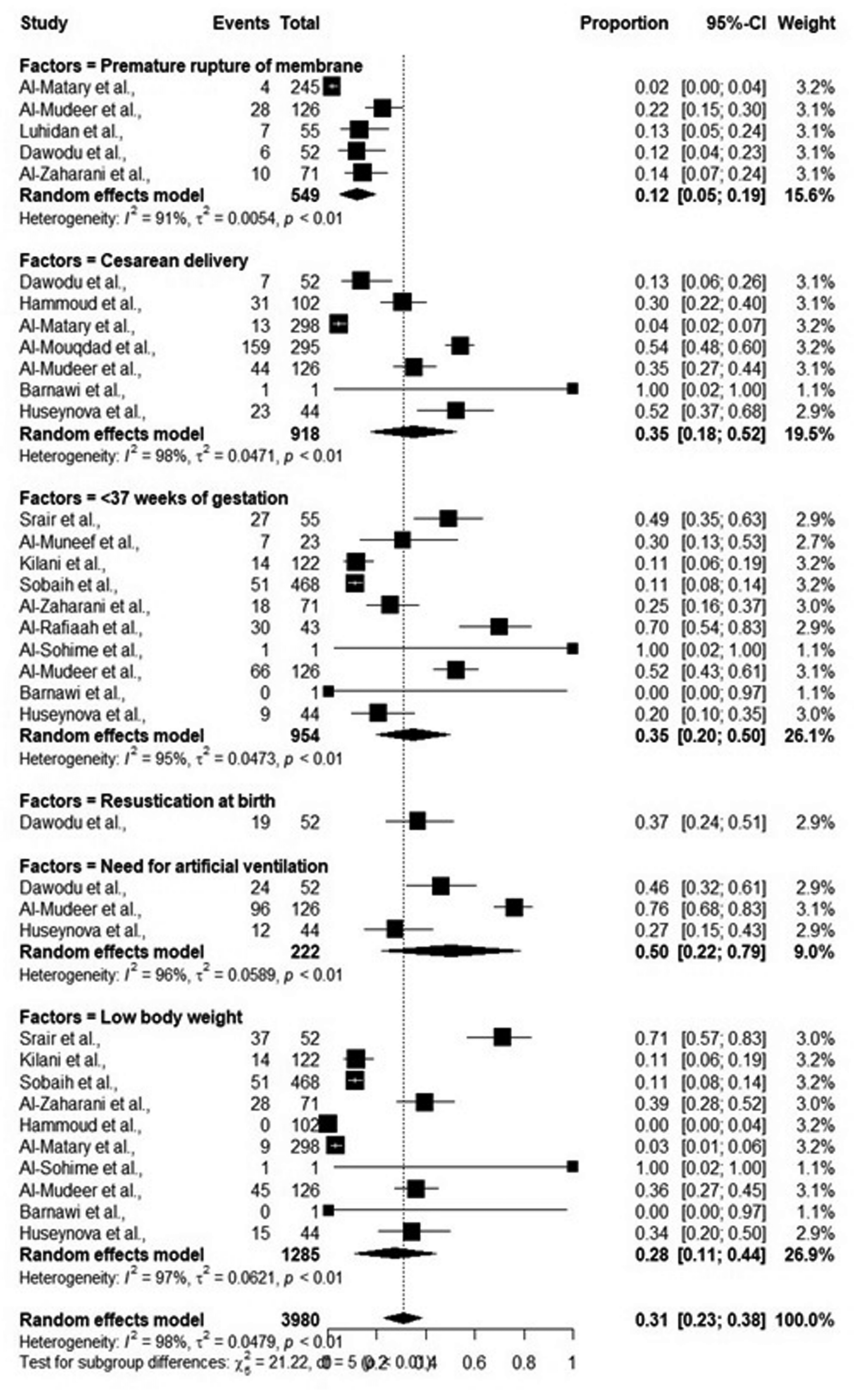
Foster plot.

While performing meta-analysis, significant heterogeneity between included studies, as shown by *I*^2^ (>90%) was observed, thus all the data were analyzed following random effect models. Overall, the risk factors including mother-related and neonate-related increased the chances of early onset sepsis (EOS). Most of the studies reported that pre-term neonates (95% CI: 0.20–0.50) and low birth weight neonates (95% CI: 0.11–0.44) were at elevated risk of EOS. The detailed results are shown in Table 3 and Figure 4.

**Table 3:** Risk factors included in meta-analysis of early onset sepsis.

Risk factors	No. of studies	Sample size	Weight (%)	95% confidence interval		Heterogeneity *I*^2^	*P*-value
				LCI	UCI		
Mother-related							
Premature rupture of membrane	5	549	15.6%	0.05	0.19	91%	<0.01
Cesarean delivery	7	918	19.5%	0.18	0.52	98%	<0.01
<37 weeks of gestation	10	954	26.1%	0.20	0.50	95%	<0.01
Neonate-related							
Low birth weight	10	1,285	26.9%	0.11	0.44	97%	<0.01
Resuscitation at birth	1	52	2.9%	0.24	0.51	Not estimated	Not estimated
Need of artificial ventilation	3	222	9%	0.22	0.79	96%	<0.01

### 3.3. Quality assessment

The quality of the included studies was assessed using the Newcastle-Ottawa Scale (NOS) for cohort studies and Joanna briggs Institute (JBI) critical checklist for case study. For cohort studies, the NOS was used to assess the methodological quality of each study. This scale evaluates the risk of bias in three domains: selection of study groups, comparability of groups, and the ascertainment of exposure/outcome. Each study is awarded a maximum of nine stars based on its quality in these three domains, with higher scores indicating lower risk of bias. This checklist includes criteria such as clear identification of the problem, description of the context, adequate data analysis, and appropriate conclusions. Each study was evaluated against these criteria to determine its methodological quality any discrepancies between the reviewers were resolved through discussion and consensus. The details are mentioned in Tables 4, 5.

**Table 4:** Quality assessment of cohort studies.

Reference	Selection				Comparability	Outcomes		Quality Score
	Representative of sample^a^	Sample size^b^	Non-respondents^c^	Ascertainment of exposure^d^	Comparability of cohort studies on basis of design^e^	Assessment of outcomes^f^	Statistical analysis^g^	
Al-Matary et al., 2019 (27)	*	*	–	–	*	**	*	6
Al-Mouqdad et al., 2018 (29)	*	*	–	**	*	**	*	8
Al-Rafiaah et al., 2016 (30)	*	*	–	–	*	**	*	6
Al-Zaharani et al., 2015 (31)	*	*	–	*	*	**	*	7
Almuneef et al., 2000 (32)	*	*	–	**	*	**	*	8
Hammoud et al., 2017 (28)	*	*	–	*	*	**	*	7
Kilani et al., 2000 (33)	*	*	–	–	*	**	*	6
Huseynova et al., 2021 (34)	*	*	–	**	*	**	*	8
Al-Mudeer et al., 2020 (35)	*	*	–	–	*	**	*	6
Luhidan et al., 2019 (36)	*	*	–	**	*	**	*	8
Sobaih et al., 2014 (37)	*	*	–	*	*	**	*	7
Nasr et al., 2013 (38)	*	*	–	*	*	**	*	7
Fattah et al., 2017 (39)	*	*	–	*	*	**	*	7
Dawodu et al., 1997 (40)	*	*	–	*	*	**	*	7
Haque et al., 1990 (42)	*	*	–	–	*	**	–	5
Ohlsson et al., 1986 (43)	*	*	–	–	*	**	–	5
Elbashier et al., 1994 (45)	*	*	–	*	*	**	*	7
Abu-Osba et al., 1989 (46)	*	*	–	–	*	**	*	6
Srair et al., 1993 (47)	*	*	–	–	*	**	*	6

^a^ * = truly representative or somewhat representative of average in target population.

^b^ * = Drawn from the same community.

^c^ * = Secured record or structured review.

^d^ * = Yes, − = No.

^e^ * = Study controls for age, gender, and other factors.

^f^ * = Record linkage or blind assessment, ** = Both.

^g^ * = follow-up of all subjects.

**Table 5:** Quality assessment of case reports.

Study	Q1	Q2	Q3	Q4	Q5	Q6	Q7	Q8	Quality rating
Dawodu et al., 1997 (40)	Yes	Yes	Yes	Yes	Yes	Yes	No	Yes	Good
Barnawi et al., 2020 (41)	Yes	Yes	Yes	Yes	Yes	Yes	Yes	Yes	Good

Q1. Were patient's demographic characteristics clearly described? Q2. Was the patient's history clearly described and presented as a timeline? Q3. Was the current clinical condition of the patient on presentation clearly described? Q4. Were diagnostic tests or assessment methods and the results clearly described? Q5. Was the intervention(s) or treatment procedure(s) clearly described? Q6. Was the post-intervention clinical condition clearly described? Q7. Were adverse events (harms) or unanticipated events identified and described? Q8. Does the case report provide takeaway lessons?

## 4. Discussion

This systematic review is one of the broadest studies to show the pattern, risk and diagnostic factors, and clinical outcome of newborn with NEOS from Saudi Arabia. Findings of 21 studies were included which are consistent with observations from other research indicating the proportion of NEOS is significantly higher (48, 49). The risk factors identified are similar to other previous studies, focusing on the significance of premature rupture of membrane, premature birth, and low birth weight. According to a study, NEOS is more common in newborns with extremely low birth weights, decreased respiratory function at delivery, and mother related risk factors (50). These results may be explained by preterm babies' weak innate immune system, which increase their risk of developing NEOS (5). NEOS develops in the uterus as a result of maternal blood infection or, more frequently because of infection of the placenta and the amniotic fluid (51). Patients may present with fetal distress, pneumonia, newborn asphyxia or sepsis owing to aspiration of contaminated amniotic fluid or secretions after delivery (52). A comprehensive study looked at whether newborn clinical presentation might be used to rule out EOS in babies born to moms who had chorioamnionitis. According to the investigation, EOS can happen in infants who had initially positive clinical status (53). A range of services are available in different healthcare settings included in this review, to manage high-risk newborns who are prone to infection. Nevertheless, in our review, we discovered a variety of variables, including a low Apgar score, in a sizable number of neonates as a risk factor for NEOS. This finding is consistent with other studies that revealed a low Apgar score to be strongly related with newborn sepsis (48, 49, 54). Low Apgar ratings in newborns make them more vulnerable to infection because they are less able to handle stress from outside sources (55). Furthermore, the findings revealed that NEOS patients had low birth weights, which is consistent with a finding from other research (56). The leading maternal risk factors for NEOS were found to be multiparty and caesarean birth. This was also the main maternal risk factor for NEOS according to a research by Al Dasoky et al. in a tertiary care hospital in Jordan (57). A valuable measure of the health of the newborn and the severity of the illness is the total WBC count. Our review revealed that the patients' WBC counts were disturbed which is a sign of a severe illness in the newborn (58). The fact that NEOS occurs while the mother and child are still confined to the hospital and that there is a significant risk of hospital acquired infection (59).

In developed and underdeveloped countries, different pathogens are responsible for causing newborn sepsis including both Gram-negative and positive bacteria (60). Nonetheless, the present initiatives for maternal intrapartum antibiotic prophylaxis against GBS may be responsible for the more prevalence of Gram-negative microbes (61). A variety of pathogens are responsible for neonatal EOS, depending on multiple factors, such as hospital practices, geographical region, and changes in microbial resistance pattern. Overall, Group B streptococcus and E. coli are the leading pathogens for EOS. However, the patterns of pathogens may vary from state to state. According to our findings, GBS was the most prevalent etiologic agent in NEOS, followed by E. coli. Our study reported that *Staphylococcus* species are responsible for EOS in Khobar, *E. coli* in Jazan and *Klebsiella* spp. in Taif. Stoll et al., reported that *E. coli* associated sepsis was increased from 3.2 to 6.8 per 1,000 live births. Likewise, the most frequently identified agent in NEOS in European countries is GBS, followed by Gram-negative bacilli and staphylococci (62). Moreover, GBS, staphylococci, gram-negative bacteria, and candida were identified in NEOS patients in other regions as well (6, 7, 9, 16, 49). According to earlier research that is in line with our results, infant sepsis mortality is also very high and had a mortality rate for NEIOS 10%–20% (63). Neonates are more susceptible to infections in ICU settings because of their immature immune system, and exposure to invasive medical procedures.

To the best our knowledge, this is first systematic review carried out from the KSA and is notable because it represents a comprehensive complete database evaluated to identify the pattern, diagnostic and risk factors, and therapy outcomes of NEOS in different healthcare institutes in KSA. It is crucial to undertake more controlled research studies since neonatal safety is so important. Incidence or prevalence of NEOS in the KSA population must be quantified. Moreover, larger trials are necessary to assess the safety and effectiveness of antibiotic therapy. However, no adverse drug reactions were seen, indicating that even if a negative effect existed, it would likely be rare and would need to be considered in relation to the unfavourable consequences of sepsis assessments and antibiotic exposure. Prior research studies from other parts of world have demonstrated the need of clinical monitoring as a component of any NEOS approach by showing that GBS-specific NEOS persists in children delivered to moms who screen erroneously negative for GBS without additional intrapartum risk factors for EOS (64–66). Nonetheless, this systematic review provides a valuable baseline data to start in KSA. Future studies could explore further questions.

## 5. Conclusions

This systematic review highlighted the various substantial risk factors associated with development of neonatal early onset sepsis in ICU patients. The prevalence of EOS is high in neonates admitted to ICU with multiple maternal and neonatal risk factors. Therefore, by identifying such factors, the healthcare experts can implement targeted preventive strategies and monitor these risk factors, thus reducing the prevalence of EOS and improving the therapeutic outcomes. Moreover, high-quality studies and better diagnostics are required especially in hospitals with high neonatal mortality to evaluate the prevalence of EOS, identify the associated risk factors and clinical outcomes.

## Data Availability

The original contributions presented in the study are included in the article/**Supplementary Material**, further inquiries can be directed to the corresponding author.
