# Supplementary material for: Risk and diagnostic factors and therapy outcome of neonatal early onset sepsis in ICU patients of Saudi Arabia: a systematic review and meta analysis
Source: Front Pediatr. 2023 Aug 23;11:1206389. doi: 10.3389/fped.2023.1206389 (PMC10482413; doi:10.3389/fped.2023.1206389)
Supplement: Supplementary file 1 [file Table1.docx]

# Table S1. Retrieval strategy and search results from Databases

| **#** | **Search terms** | **Results** |
| --- | --- | --- |
| 1 | EARLY ONSET SEPSIS [MeSH Terms] OR EARLY ONSET SEPSIS [Text Word] OR early onset sepsis [MeSH] OR early onset sepsis [Text Word] | 163,432 |
| 2 | NEONATES [MeSH Terms] OR Neonates [Text Word] OR Pediatrics [MeSH Terms] OR Pediatrics [Text Word] | 121,543 |
| 3 | Risk Factors [MeSH Terms] OR Maternal Factors *[Text Word] OR Neonatal Factors* [Text Word] | 89,756 |
| 4 | Diagnostic Factors [MeSH Terms] OR Diagnostic Factors [Text Word] OR Diagnosis [MeSH Terms] OR Diagnosis [Text Word] | 76,236 |
| 5 | Therapeutic Outcomes [MeSH Terms] OR Therapeutic Outcomes [Text Word] OR Clinical Outcomes [MeSH Terms] OR Clinical Outcomes [Text Word] | 77,794 |
| 6 | #1 AND #2 AND #3 | 57,895 |
| 7 | #1 AND #2 AND #4 | 16,564 |
| 8 | #1 AND #2 AND #5 | 21,421 |
| 9 | #1 AND #2 AND #3 AND #4 AND #5 | 12,345 |
